# Supplementary material for: Interaction-based Mendelian randomization with measured and unmeasured gene-by-covariate interactions
Source: PLoS One. 2022 Aug 10;17(8):e0271933. doi: 10.1371/journal.pone.0271933 (PMC9365161; doi:10.1371/journal.pone.0271933)
Supplement: S1 Table — (PDF) [file pone.0271933.s001.pdf]

Table 1: **Simulated results and effect estimates for subset of interaction (denoted Z) identified from Fig 4A (simulation 1). Estimates represent mean values across 1,000 independent data sets.**

Interaction covariate	Estimate	95% CI	F-statistic (P-value)
Z_{14}	1.000	(0.995-1.004)	2515.92 (< 0.001)
Z_{24}	1.000	(0.995-1.004)	3056.58 (< 0.001)
Z_{51}	1.000	(0.995-1.005)	2463.58 (< 0.001)
Z_{58}	1.000	(0.995-1.005)	2252.72 (< 0.001)
Z_{75}	1.000	(0.995-1.005)	2408.85 (< 0.001)
Z_{80}	1.000	(0.995-1.005)	1852.88 (< 0.001)
Z_{88}	1.000	(0.995-1.005)	2314.96 (< 0.001)
Z_{90}	1.000	(0.995-1.004)	2172.93 (< 0.001)
Z_{92}	1.000	(0.996-1.004)	2653.29 (< 0.001)
Z_{93}	1.000	(0.996-1.004)	2681.99 (< 0.001)
